# Integrative population genetics and multi-omics reveal IL15RA as a shared pathogenic hub in rheumatoid arthritis-related heart failure

**DOI:** 10.3389/fimmu.2026.1956897

**Published:** 2026-10-12

**Authors:** Qishun Geng, Chaoying Wen, Meijia Yang, Yuhan Zhou, Meng Yang, Qishun Geng, Jie Zhao

**Affiliations:** 1 Department of Pharmacy, The First Affiliated Hospital of Zhengzhou University, Zhengzhou, Henan, China; 2 Internet Medical and System Applications of National Engineering Laboratory, The First Affiliated Hospital of Zhengzhou University, Zhengzhou, Henan, China; 3 China-Japan Friendship Clinical Medical College, Chinese Academy of Medical Sciences & Peking Union Medical College, Beijing, China; 4 Institute of Clinical Medical Sciences, China-Japan Friendship Hospital, Beijing, China; 5 Department of Oncology, The First Affiliated Hospital of Zhengzhou University, Zhengzhou, Henan, China; 6 Department of Orthopedics, Henan Provincial Third People’s Hospital, Zhengzhou, Henan, China

**Keywords:** dioscin, heart failure, IL15RA, inflammation, rheumatoid arthritis

## Abstract

**Purpose:**

Rheumatoid arthritis (RA) patients face a significantly elevated risk of heart failure (HF), but population-level evidence and underlying mechanisms remain unclear. We aimed to assess the RA-HF association using national data and identify dietary compounds targeting shared inflammatory pathways.

**Methods:**

We analyzed NHANES data (n=2,931) and performed two-sample Mendelian randomization (MR). Integrated machine learning, single-cell transcriptomics, clinical samples, and *in vitro* experiments identified therapeutic targets and evaluated the effectiveness of dioscin (a yam-derived saponin).

**Results:**

The rate of HF in RA patients is high, and RA patients with HF have a worse prognosis. MR analysis also revealed that RA was a significant HF risk factor. IL15RA emerged as a shared causal target, whose silencing suppressed TNF-α-induced ICAM-1 in endothelial cells and reduced M1 (CD86^+^) macrophage polarization. Mechanistically, MIF signaling were strongly implicated in the crosstalk between endothelial cells and macrophages. Dioscin bound IL15RA and reduced its expression in both cell types, phenocopying IL15RA silencing.

**Conclusion:**

This study identifies IL15RA as a key therapeutic target linking RA and HF, and dioscin as a dietary phytochemical with anti-inflammatory potential. Our findings provide a population-based framework and a readily translatable nutritional strategy for mitigating cardiac comorbidity in RA patients.

## Introduction

Rheumatoid arthritis (RA) is a systemic autoimmune disease, which can lead to progressive joint destruction and severe disability (1). However, the impact of RA extends far beyond the joints, and cardiovascular disease (CVD) is the leading cause of premature death in the RA population (2). Among of them, heart failure (HF) is a particularly severe endpoint, with RA patients exhibiting a significantly elevated risk (3, 4). This epidemiological link highlights a fundamental pathophysiological connection: chronic systemic inflammation is a common cause of both joint destruction and cardiac dysfunction (5). Although targeted immunomodulatory therapies have advanced, improving the prognosis of RA, their ability to fully mitigate cardiovascular risk remains unsatisfactory (6). This critical therapeutic gap highlights the urgent need to identify common inflammatory pathways that drive both RA and HF.

The pathogenesis of HF in patients with RA is multifactorial, including accelerated atherosclerosis, myocardial ischemia, and myocarditis. The core of these processes involves the activation of vascular endothelial and innate immune cells, maintained by the proinflammatory environment of RA (7, 8). Once activated, endothelial cells upregulate the expression of adhesion molecules (such as VCAM-1 and ICAM-1) and secrete chemokines, promoting the infiltration of inflammatory cells into synovial and vascular tissues. This persistent endothelial dysfunction is the foundation for atherosclerotic plaque formation and impaired coronary microvascular function (9). Moreover, macrophages, as key coordinators of inflammation and tissue remodeling, exacerbate synovitis and bone erosion (10). In the cardiovascular system, macrophages cause plaque instability and promote myocardial fibrosis (11). Therefore, identifying the common inflammatory signaling pathways in macrophages and endothelial cells is crucial. IL-15RA can effectively activate synovial macrophages, promoting the production of inflammatory factors such as TNF-α (12). Additionally, the IL-15/IL-15RA axis directly activated endothelial cells, induce the expression of adhesion molecules, and promote angiogenesis, a process associated with synovial inflammation and the progression of atherosclerotic plaques (13, 14). Therefore, IL-15RA is a shared target of the inflammatory cascade in endothelial cells and macrophages.

Dietary bioactive compounds have great potential in disease treatment; Dioscin, as a steroidal saponin present in edible plants of the Dioscorea genus, exhibits a broad range of biological activities, including anti-inflammatory, antioxidant, and cardioprotective properties (15). Preclinical studies have indicated that dioscin alleviated collagen-induced arthritis (CIA) in rats by inhibiting the production of proinflammatory cytokines and modulating immune cell function (16). Additionally, in cardiovascular injury models, dioscin has been shown to protect against myocardial ischemia-reperfusion injury, atherosclerosis, and doxorubicin-induced cardiotoxicity, primarily because of its anti-inflammatory and antiapoptotic properties (17). Mechanistic exploration suggested that dioscin may interfere with key signaling hubs such as the NF-κB and MAPK pathways (18). However, in the context of inflammatory diseases, the precise molecular mechanism of dioscin remains elusive.

This study revealed that HF exacerbates the poor prognosis of RA and that there is a causal relationship between RA and HF. Furthermore, IL15RA plays a significant regulatory role in the progression of both RA and HF. And dioscin can inhibit the expression of IL15RA in endothelial cells and macrophages, effectively alleviating synovial and cardiovascular inflammation. Our findings provide a population-based framework and a readily translatable nutritional strategy for mitigating cardiac comorbidity in RA patients.

## Methods

### Data source and study population

In this study, data from the NHANES collected between 1999 and 2018 were used. We excluded (1) participants<20 years (n=46235), (2) participants without RA (n=52129), (3) participants with missing mortality data (n=5), and (4) participants with missing HF data (n=16). The final sample of participants included in this analysis was 2931. This self-reported diagnostic approach was used to identify patients of RA and HF (19, 20). All regression analysis involving NHANES data properly incorporated a complex survey design using survey-weighted regression.

The GSE5406 and GSE89408 datasets were used as training sets. The data from GSE12021, GSE55235, GSE55457 and GSE55584 were retrieved from the Affymetrix^®^ GPL96 platform, which were merged as a test set for the RA predictive model. GSE116250 dataset was used as a test set for the HF predictive model (**Supplementary Table 1**). We implemented rigorous batch effect correction using the ComBat algorithm from the sva package.

### Cell culture and treatments

RAW264.7 cells and HUVECs were obtained from the Chinese Academy of Sciences (Shanghai, China). The siIL15RA target sequences are shown below: Human siIL15RA, 5’- GCUUCAUCUCCCAGCUCAAAC-3’. Mouse siIL15RA, 5’- GGGAGAGGUAUGUCUGUAACU-3’. JetPRIME^®^ Transfection Reagent was used for cell transfection (Polyplus Transfection, USA).

### Histopathological analysis and immunofluorescence analysis

The synovial tissues of the RA patients and normal controls were obtained from China-Japan Friendship Hospital. Heart tissues from cardiovascular disease (CVDs) and normal controls were obtained from SHANGHAI OUTDO BIOTECH Co., Ltd. (N0025G0100 and N0026G0100, Shanghai, China). Samples processing and data analysis were performed as previously described (21). The JACoP plugin of ImageJ was used for fluorescence colocalization analysis to calculate the Pearson correlation coefficient (PCC) (22).

### Real-time PCR analysis and western blotting

In accordance with previously described methods, we processed the samples and analyzed data (23). The primer sequences are showed in **Supplementary Table 2**. The primary antibodies: anti-ICAM-1 (60299-1-lg, Proteintech), anti-IL15RA (16744-1-AP, Proteintech) and anti-GAPDH (60004-1-lg, Proteintech).

### Mendelian randomization analysis

The UKB-a-105 (RA) and ebi-a-GCST009541 (HF) datasets were acquired from the genome-wide association study (GWAS) database. SNPs (p<1e-5) were used as instrumental variables in the ukb-a-105 dataset, which was chosen as the exposure group. r^2^ < 0.001 at a distance of 10,000 kb was selected to avoid the effects of linkage disequilibrium. To ensure sufficient strength, SNPs, with F>10, were selected. Mendelian randomization effect analysis was performed to evaluate the relationship between the RA and HF. In addition, cis-eQTLs data, from eQTLGen, were used to explore the potential causal relationships among genes, RA and HF.

### Machine learning algorithms

Logistic regression, partial least squares (PLS), least absolute shrinkage and selection operator (LASSO), support vector machine with a kernel function (SVM_Kernel), random forest, generalized boosting method, gradient boosting machine, adaptive boosting, categorical boosting (CatBoost), light gradient boosting machine (LightGBM), nearest neighbor method, neural network (NeuralNet), Bayesian method, and discriminant model were used for machine learning. The caret package was used for model training and cross-validation. Hyperparameter tuning was performed on the training set using 5-fold cross-validation repeated 10 times.

### Single-cell analysis

Based on the GSE299518 and GSE222144 dataset, we conducted scRNA-seq analysis following the previous methods (24, 25). scTenifoldKnk was used for virtual gene knockout analysis based on scRNA-seq data (26).

### Statistical analysis

R version 4.0.4 software (https://www.r-project.org) and GraphPad Prism 8 software were used for statistical computing and data visualization. All the experimental procedures were conducted with three independent biological replicates. Intergroup comparisons were assessed with two-tailed Student’s t-tests. Quantitative results are reported as the mean ± standard deviation (SD), with a statistical significance threshold defined as P< 0.05.

## Results

### Prevalence and risk factors for HF in adults with RA

On the basis of the NHANES database, we included 2,931 RA patients, among whom 306 (weighted percentage 8.89%) had HF. The baseline characteristics of the participants are shown in **Supplementary Table 3**. HF patients are more likely to be over 60 years old, be non-Hispanic White, be married, and have a high school education or above, be smokers, and have hypertension, diabetes, kidney failure or stroke (all p values < 0.05).

Afterwards, we further compared the odds ratios for HF in RA patients with different characteristics (**Supplementary Table 4**). Both univariate and multivariate analyses indicated that being over 60 years old, having an education level lower than high school; being a non-Hispanic White individual; being a smoker; and having a history of hypertension, diabetes, stroke, and kidney failure were risk factors for HF. Therefore, the risks of HF were as follows: age >60 years (OR 2.5, 95% CI [1.68–3.73]); non-Hispanic White ethnicity (OR 2.03, 95% CI [1.15–3.56]); a high school education level or above (OR 0.7, 95% CI [0.49–1]); smoking (OR 1.49, 95% CI [1.05–2.1]); and a history of hypertension (OR 1.83, 95% CI [1.18–2.83]), diabetes (OR 1.91, 95% CI [1.33–2.73]), stroke (OR 3.02, 95% CI [1.87–4.87]), and kidney failure (OR 1.922, 95% CI [1.16–3.18]).

### Association of HF with all-cause mortality in adults with RA

Considering the high incidence of HF in RA patients, we further analyzed the impact of HF on the survival rate of patients. We constructed three models. Model 1 did not correct for any covariates, Model 2 corrected for only age, gender, marital status, income status, education and race, and Model 3 corrected for all 14 covariates. Using people without HF as a reference, Cox regression was used to analyze the correlation between HF and all-cause mortality. The results showed that when covariates were not adjusted, compared with people without HF, people with HF had an increased risk of all-cause mortality. The HR was 2.88 (95% CI: 2.28-3.64). When adjusting for the effects of age, gender, marital status, income status, education and race, compared with those who did not have HF, people with HF had an increased risk of all-cause mortality. The HR was 1.86 (95% CI: 1.52-2.27). When all 14 covariates were adjusted, people with HF had an increased risk of all-cause mortality (HR 1.56, 95% CI [1.29-1.88]) (**Table 1**). The events-per-variable (EPV) ratio was calculated to assess the risk of overfitting; EPV (68.24) ≥ 10 was considered adequate (27). These findings suggest that HF is a risk factor for all-cause mortality in adults with RA.

**Table 1:** Hazard ratios for all-cause mortality in RA patients with HF.

Characteristics	Model	Hazard ratio	CI_low	CI_high	*P*-value
Heart failure	Model 1	2.88	2.28	3.64	*P* < 0.001
	Model 2	1.86	1.52	2.27	*P* < 0.001
	Model 3	1.56	1.29	1.88	*P* < 0.001

Reference: Model 1 did not correct for any covariates, Model 2 corrected for only age, gender, marital status, income status, education level and race, and Model 3 corrected for all 14 covariates.

### The correlation between RA and HF and the key targets were determined through Mendelian randomization analysis

Firstly, two-sample Mendelian randomization analysis was performed with the ebi- ukb-a-105 (RA) and ebi-a-GCST009541 (HF) datasets to compare the relationship between the RA and HF, using 23 SNPs. All F-statistic values exceeded 10, indicating minimal likelihood of weak instrument bias. The results of inverse variance weighted (IVW) analysis revealed a relationship between RA and HF (OR = 14.8783, p<0.05) (**Figures 1A, B**). There was a strong positive correlation between genetic susceptibility to RA and HF. To assess the robustness of the results, several sensitivity analysis were conducted. Heterogeneity was not observed (Cochrane’s Q = 32.36, p=0.071). Importantly, there was no evidence of directional pleiotropic effects on genetic variation, which was revealed by MR-Egger regression (Intercept =0.0051, Se = 0.0045, p = 0.27, **Figure 1C**). Similarly, the MR-PRESSO results indicated no horizontal multidirectionality in this MR analysis (RSSobs =41.83424, p=0.077). The MR-RAPS estimate remained consistent with the primary IVW result (β = 2.7906, SE = 1.0174, p = 0.0061; overdispersion χ² = 32.11). The leave-one-out sensitivity analysis indicated that the relationship between RA and HF was not attributable to a single SNP (**Figure 1D**). The reverse Mendelian randomization analysis argues strongly against reverse causation (IVW OR = 1.0, 95% CI [0.99-1.00], p = 0.72). These findings supported the findings in our analysis. Overall, RA is a risk factor for HF occurrence.

**Figure 1:**
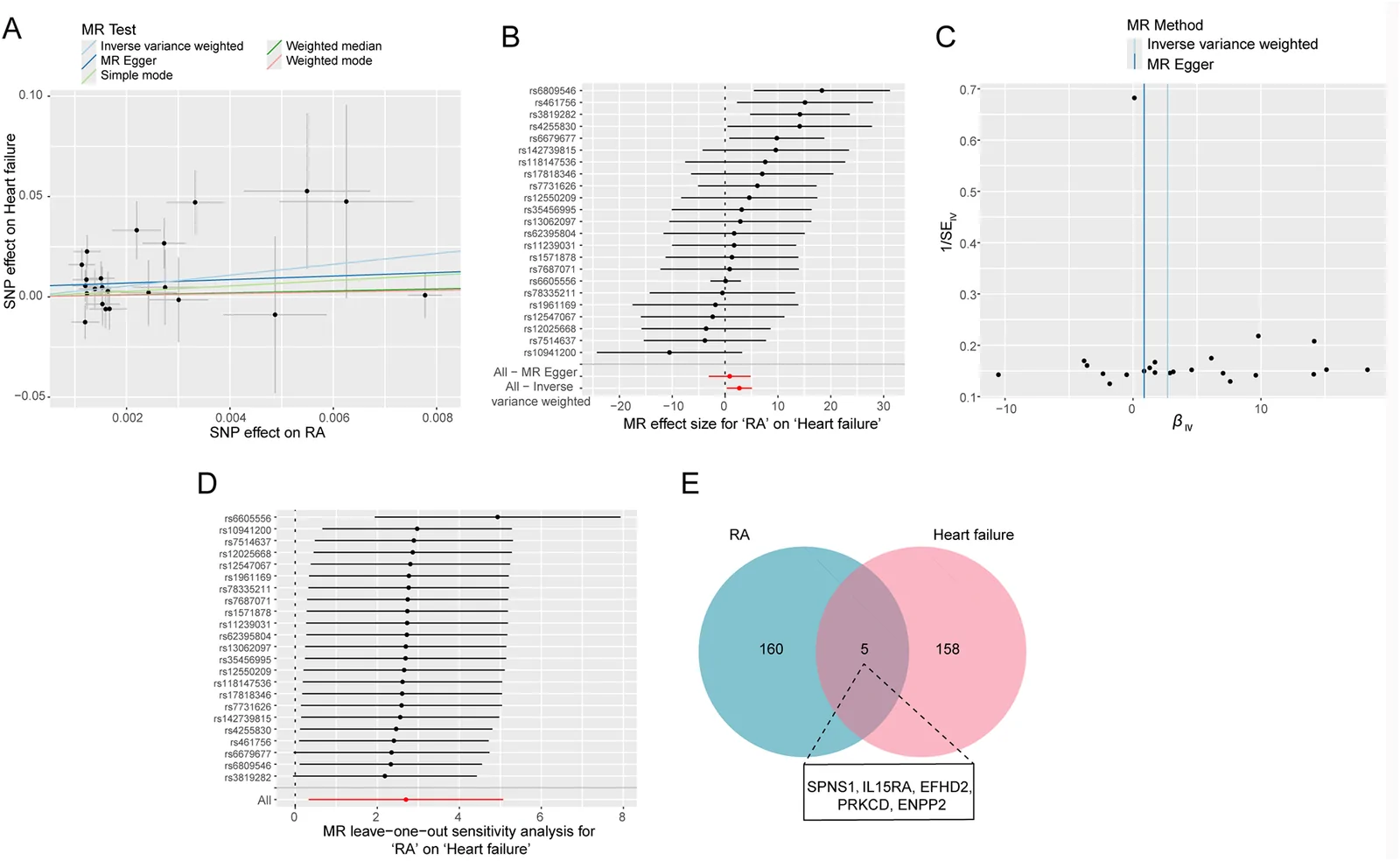
RA is a risk factor for HF. Scatter plot **(A)** and forest plot **(B)** of the effects of RA-associated SNPs on HF. **(C)** Funnel plot of the role of RA-associated SNPs on HF. **(D)** Leave-one-out plots for the Mendelian randomization analysis of RA on HF. **(E)** The Venn diagram of the intersection gene that have causal relationships with both RA and HF.

In order to determine the key targets in RA and HF, using cis-eQTLs data from eQTLGen (28), we conducted a two-sample MR study to explore the potential causal relationships among genes, RA and HF. MR-Egger regression and leave-one-out analysis were used to ensure that there was no heterogeneity or pleiotropy. We found that 165 genes have a causal relationship with RA and that 163 genes have a causal relationship with HF. Among them, 5 genes are causally related to both RA and HF, namely SPNS1, EFHD2, PRKCD, IL15RA and ENPP2 (**Figure 1E**).

### IL15RA is an important therapeutic target for RA patients with HF

Based on the expression levels of 5 intersecting genes, a disease prediction model (RA vs. non- RA) was constructed using 14 machine learning methods. All 14 models were trained on the training set with 10-repeated 5-fold cross-validation for hyperparameter tuning (**Figure 1A**). To avoid overfitting in internal validation, the independent test set was then used to evaluate and select the best-performing model (**Figure 2B**). Among of them, the Logistic models had the highest AUC (0.878), which was selected for SHAP value interpretation. The SHAP plot shows the importance of each feature in the Logistic model for predicting RA (**Figure 2C**). SHAP values indicated that IL15RA (0.146), SPNS1 (0.128) were the major contributors.

**Figure 2:**
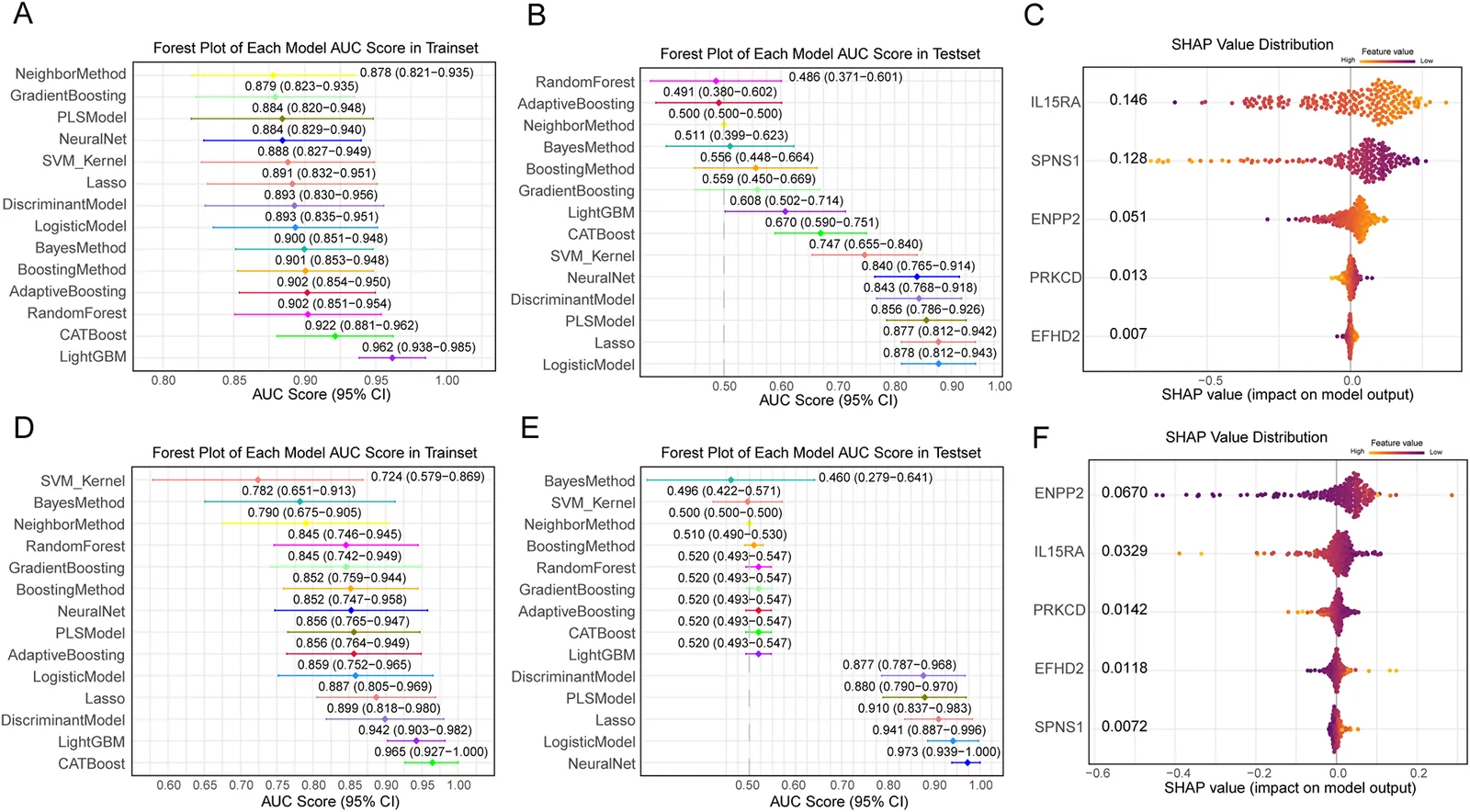
The 14 machine learning models for predicting RA and HF. **(A)** Forest plot of each model’s AUC value for predicting RA in the training set. **(B)** Forest plot of each model’s AUC value for predicting RA in the test set. **(C)** The SHAP value distribution of each feature in the logistic model for predicting RA. **(D)** Forest plot of each model’s. AUC value for predicting HF in the training set. **(E)** Forest plot of each model’s AUC value for predicting HF in the test set. **(F)** The SHAP value distribution of each feature in the NeuralNet model for predicting HF.

In addition, we constructed a disease prediction model for HF based on the transcriptome data of 5 intersecting genes. All 14 models were trained on the training set with 10-repeated 5-fold cross-validation for hyperparameter tuning (**Figure 2D**). To avoid overfitting in internal validation, the independent test set was then used to evaluate and select the best-performing model. Among them, the Logistic, NeuralNet, and Lasso models had excellent classification effects in the test set, whose AUC values were greater than 0.9 (**Figure 2E**); the NeuralNet model had the highest AUC (0.973), which was selected for SHAP value interpretation. The importance of each feature in the NeuralNet model for predicting HF showed that ENPP2 (0.067), and IL15RA (0.0329) were the key contributors. Therefore, considering the importance of genes in the RA and HF prediction models comprehensively, IL15RA is an important therapeutic target for adults with RA and HF.

### IL15RA participated in the function regulating of macrophages and endothelial cells in RA and HF

To determine IL15RA’s function, we analyzed the single-cell data to identify the role of IL15RA in different cell types.

Knee synovial tissues from RA patients were subjected to scRNA-seq analysis. Twelve cell clusters were obtained by UMAP analysis (**Figure 3A**). MonacoImmuneData and CellMarker were used to identify 5 cell types: B cell, Fibroblast, Macrophage, T cells and Monocyte (**Figure 3A**). Notably, IL15RA was particularly significant expression in macrophage and monocyte (**Figures 3B, C**). And CD86 and TNF expression was also higher in macrophage and monocyte, indicating that they are primarily M1 macrophages (**Figure 3C**). Co-expression analysis also revealed a greater percentage (9.3%) of macrophages coexpressing both IL15RA and TNF (**Figure 3D**), which indicated that IL15RA may be involved in the M1 macrophage polarization. After virtual knocking out IL15RA in macrophage, significantly changes occurred in genes such as FN1, CRTAC1, PRG4, TIMP2 and GLU (**Figures 3E, F**), which mainly participate in lymphocyte mediated immunity, B cell mediated immunity, regulation of ERK1 and ERK2 cascade and humoral immune response (**Figure 3G**).

**Figure 3:**
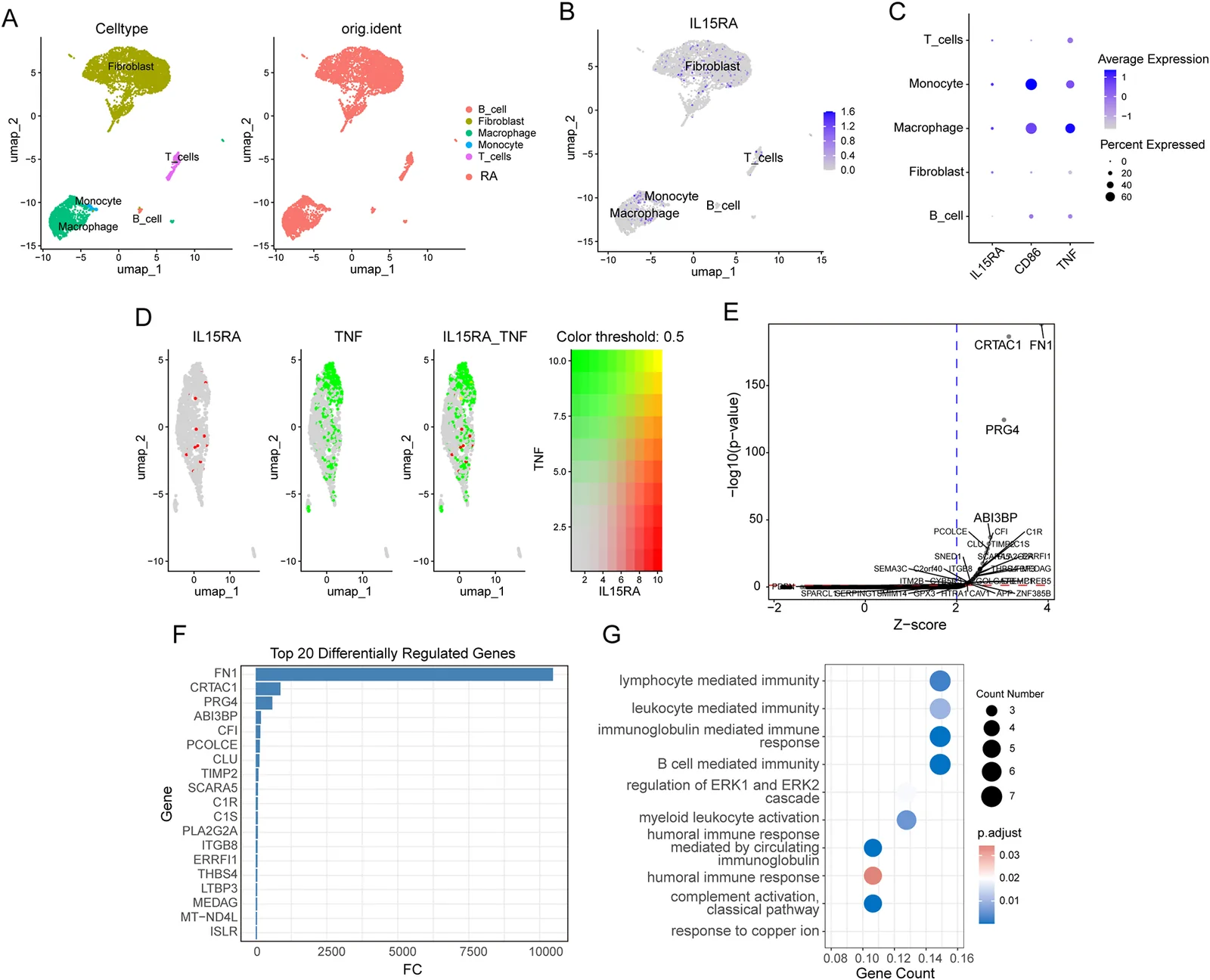
Relationship between IL15RA and CD86 expression in macrophages. **(A)** Cells are visualized by UMAP. The cell populations and sample groups are distinguished by color. **(B, C)** The expression levels of IL15RA, TNF and CD86 in different cell types. **(D)** scRNA-seq analysis revealed a correlation between IL15RA and TNF expression in macrophages. **(E, F)** Genes whose expression significantly changed after IL15RA virtual knockout in macrophages were simulated using the scTenifoldKnk package. **(G)** Functional enrichment analysis of the top 50 genes with significant changes.

Then, scRNA-Seq analysis on HF tissues and normal controls were performed. UMAP analysis visualized 15 cell clusters (**Figure 4A**). HumanPrimaryCellAtlasData and CellMarker were used to identify 6 cell types: B cells, monocyte, Neutrophils, T cells, Endothelial cells and Cardiomyocyte (**Figure 4A**). Compared with those in normal controls, the proportions of neutrophils and endothelial cells in HF tissues was significantly lower, while the proportion of monocyte was significantly greater (**Figure 4B**). Notably, IL15RA and ICAM1 were significant expression in endothelial cells (**Figure 4C**). Co-expression analysis also revealed a greater percentage (7.1%) of endothelial cells coexpressing both IL15RA and ICAM1 (**Figure 4D**). These results suggest that IL15RA may be involved in the inflammation of endothelial cells. After the virtual knockout of IL15RA in endothelial cells, significant changes were found in genes such as D2HGDH, DCN, COL6A2 and ABCA8 (**Figures 4E, F**). Functional enrichment analysis showed that they mainly participate in Focal adhesion, ECM−receptor interaction, Leukocyte transendothelial migration, Cell adhesion molecules and other signaling pathways, which are closely related to the progression of HF (**Figure 4G**). In endothelial cells, GSEA also indicated the activation of Cell adhesion molecule (CAM) interaction (**Figure 4H**). Moreover, there was a complex interaction network between endothelial cells and monocytes (**Figure 4I**). Among all signaling pathways, MIF signaling were strongly implicated in the crosstalk between endothelial cells and monocytes (**Figures 4J, K**). Receptor-ligand analysis further confirmed that both endothelial cells and monocytes express both MIF (the ligand) and its receptors CD74/CXCR4, indicating that the MIF signaling in the RA-HF microenvironment is not a unidirectional sender-receiver process but rather a complex, bidirectional network involving both paracrine and autocrine components (**Figure 4L**). This expression pattern suggests a bidirectional inflammatory amplifier between macrophages and endothelial cells in RA-HF comorbidity. Taken together, these findings suggest that IL15RA mainly plays a regulatory role in endothelial cells and macrophages in HF and RA.

**Figure 4:**
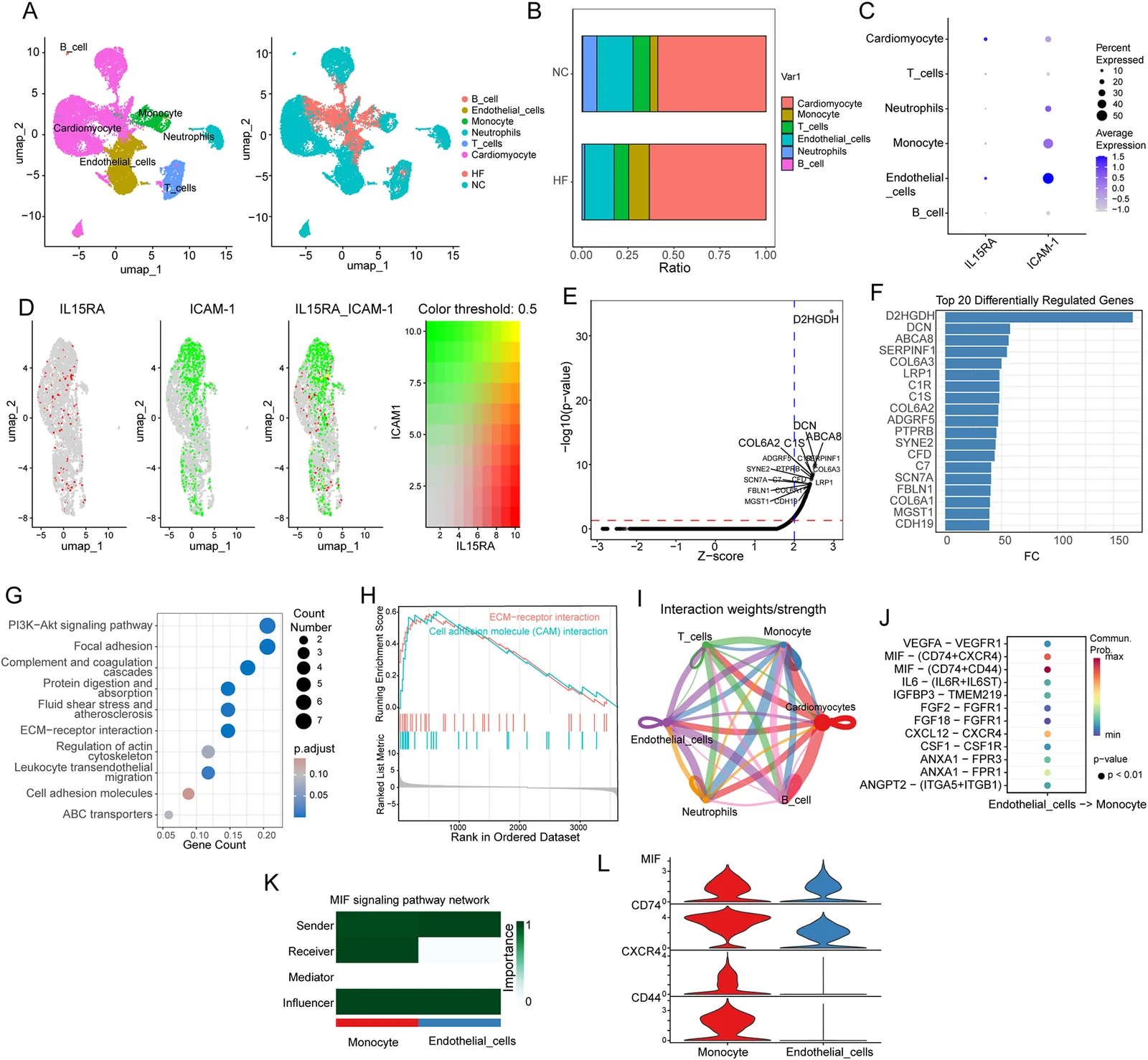
Relationships between IL15RA and ICAM-1 in endothelial cells. **(A)** Cells are visualized by UMAP. The cell populations and sample groups are distinguished by color. **(B)** The proportions of different cell types in the HF and NC groups. **(C)** The expression levels of IL15RA and ICAM-1 in different cell types. **(D)** scRNA-seq analysis revealed a correlation between IL15RA and ICAM-1 expression in endothelial cells. **(E, F)** Genes whose expression significantly changed after IL15RA virtual knockout in endothelial cells were simulated using the scTenifoldKnk package. **(G)** Functional enrichment analysis of the top 50 genes whose expression significantly changed. **(H)** GSEA analysis of endothelial cells. **(I)** Circle plot of cell–cell communications weights. **(J)** Dop plot of signaling pathways between endothelial cells and monocytes. **(K)** Heat map of MIF signaling between endothelial cells and monocytes. **(L)** The expression levels of related genes in MIF signaling.

### IL15RA is highly expressed in RA and HF, and participates in the inflammatory activation of macrophages and endothelial cells

Firstly, we examined the expression level of IL15RA in the RA synovial tissues and the cardiac tissues. The immunohistochemical results indicated that the expression level of IL15RA was higher in RA and CVDs (**Figures 5A–D**). Immunofluorescence analysis indicated that CD68 was significantly co-expressed with IL15RA in the RA synovial tissue (PCC = 0.86), which showed that IL15RA may be involved in the regulation of macrophage function (**Figure 5E**). ICAM-1 is an important adhesion molecule that mediates adhesion reactions. It expressed on vascular endothelial cells and participates in regulating inflammatory responses (29). Subsequently, we analyzed IL15RA and ICAM-1 expression in cardiac tissue, which indicated that IL15RA and ICAM-1 are highly expressed in the cardiac tissue of CVDs, and there is a co-expression relationship (PCC = 0.948, **Figure 5F**).

**Figure 5:**
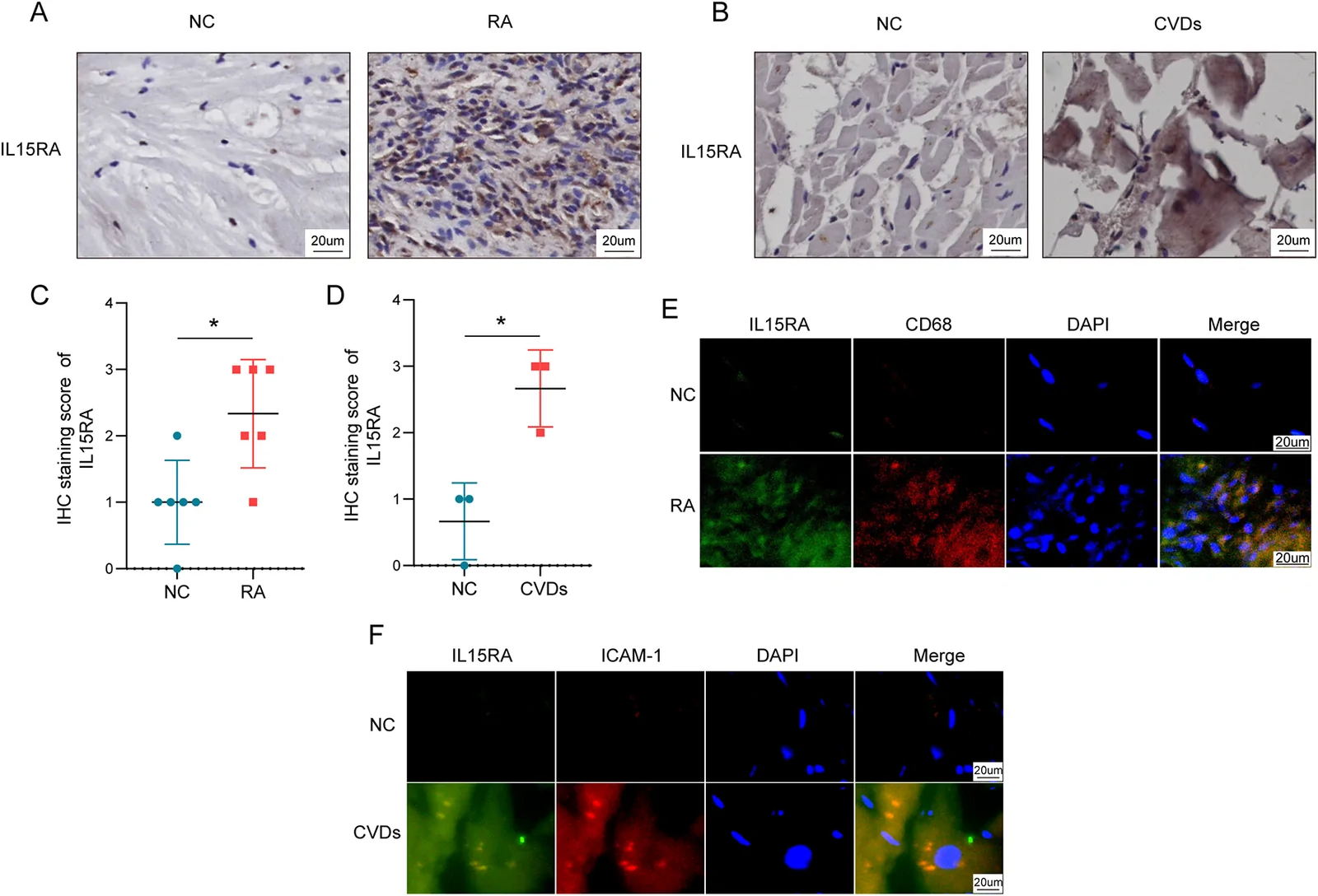
The expression levels of IL15RA, CD68 and ICAM-1 in RA and cardiovascular diseases. **(A, C)** The expression level of IL15RA was higher in the synovial tissue of RA patients. **(B, D)** The expression level of IL15RA was higher in the cardiac tissue of patients with cardiovascular diseases (CVDs). **(E)** The co-expression between IL15RA and CD68 in synovial tissue. **(F)** The co-expression between IL15RA and ICAM-1 in cardiac tissue. *p < 0.05. **p < 0.01.

To assess the relationship between IL15RA expression and inflammation, we analyzed the regulatory function of IL15RA in RAW264.7 cells and endothelial cells, respectively. The results of RT-qPCR and western blot indicated that the knockout efficiency of siIL15RA was very good in RAW264.7 cells (**Figures 6A, B**) and endothelial cells (**Figures 6D, E**). In RAW264.7 cells, siIL15RA decreased the proportion of CD86^+^ macrophages (**Figure 6C**). Besides, the western blot and IF analysis indicated that siIL15RA inhibited the upregulation of ICAM-1 expression induced by TNF-α in endothelial cells (**Figures 6E, F**). These results showed that IL15RA can be involved in regulating the inflammatory activation of macrophages and endothelial cells.

**Figure 6:**
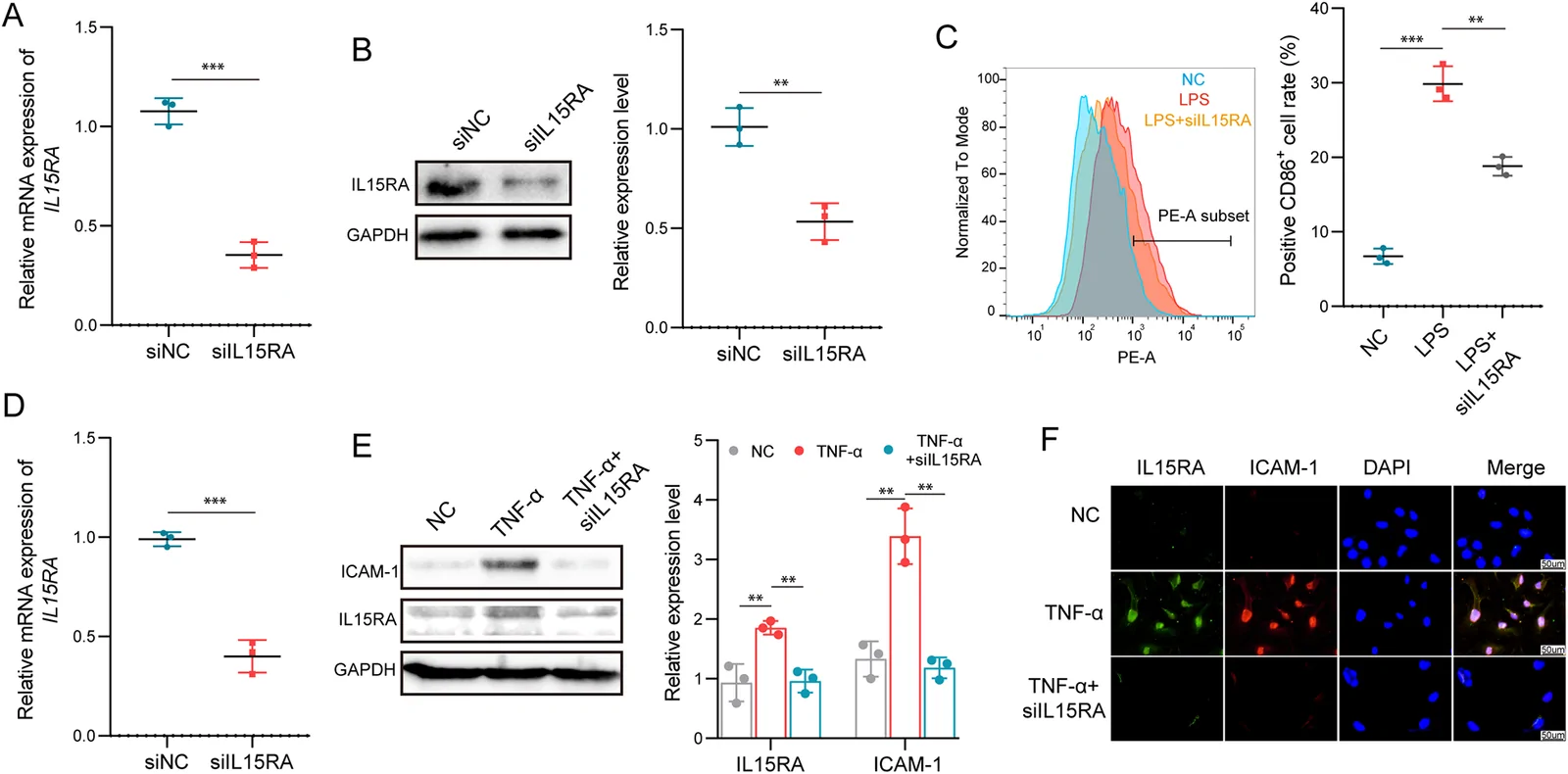
IL15RA regulates the function of macrophages and endothelial cells. The knockout efficiency of siIL15RA in RAW264.7 cells **(A)** and HUVECs **(D)**. **(B)** The expression of IL15RA in RAW264.7 cells after transfection. **(C)** The proportion of CD86^+^ macrophages. **(E)** The expression of IL15RA and ICAM-1 in HUVECs after transfection. **(F)** Immunofluorescence staining for IL15RA, ICAM-1 and DAPI in HUVECs. *p < 0.05, **p < 0.01, ***p < 0.001.

### Dioscin inhibited inflammation in macrophages and endothelial cells

Given the significant regulatory role of IL15RA in endothelial cells and macrophages, we screened a library of dietary phytochemicals to identify food-derived compounds capable of binding to the IL15RA protein. The molecular docking results indicated that the binding energy of dioscin and IL15RA was the lowest, which was -10.74 kcal/mol (**Figure 7A**). And dioscin is bound to IL15RA (PDB ID: 2Z3Q) at LEU-52, GLU-44, TYR-22, LYS-49 and HIS-55, forming 7 hydrogen bonds (**Figure 7B**). Next, we performed molecular dynamics simulations (MD) using IL15RA with dioscin to explore interactions and determine the thermodynamic stability. During the MD simulations, the stabilized range 1.6-3.2A˚ of protein-ligand interaction was observed for diosin with IL15RA in the initial 50 ns (**Figure 7C**). The fluctuation was seen between the protein-ligand complex in the trajectory between 60 and 100 ns and there was a sudden increase in the RMSD possibly due to conformational changes following initial protein-ligand interactions (**Figure 7C**). Furthermore, the stable interactions of dioscin with important amino acids GTU44 and LEU47 with ≥1 fraction interactions were visualized and retained almost 100% of stable interaction (**Figure 7D**; **Supplementary Figure S1A**). Protein-ligand contacts (number and density) of dioscin with IL15RA in each trajectory frame and root-mean square fluctuation (RMSF) graph of protein IL15RA are shown in **Supplementary Figure S1B**. These results show that dioscin possesses stable interaction with IL15RA.

**Figure 7:**
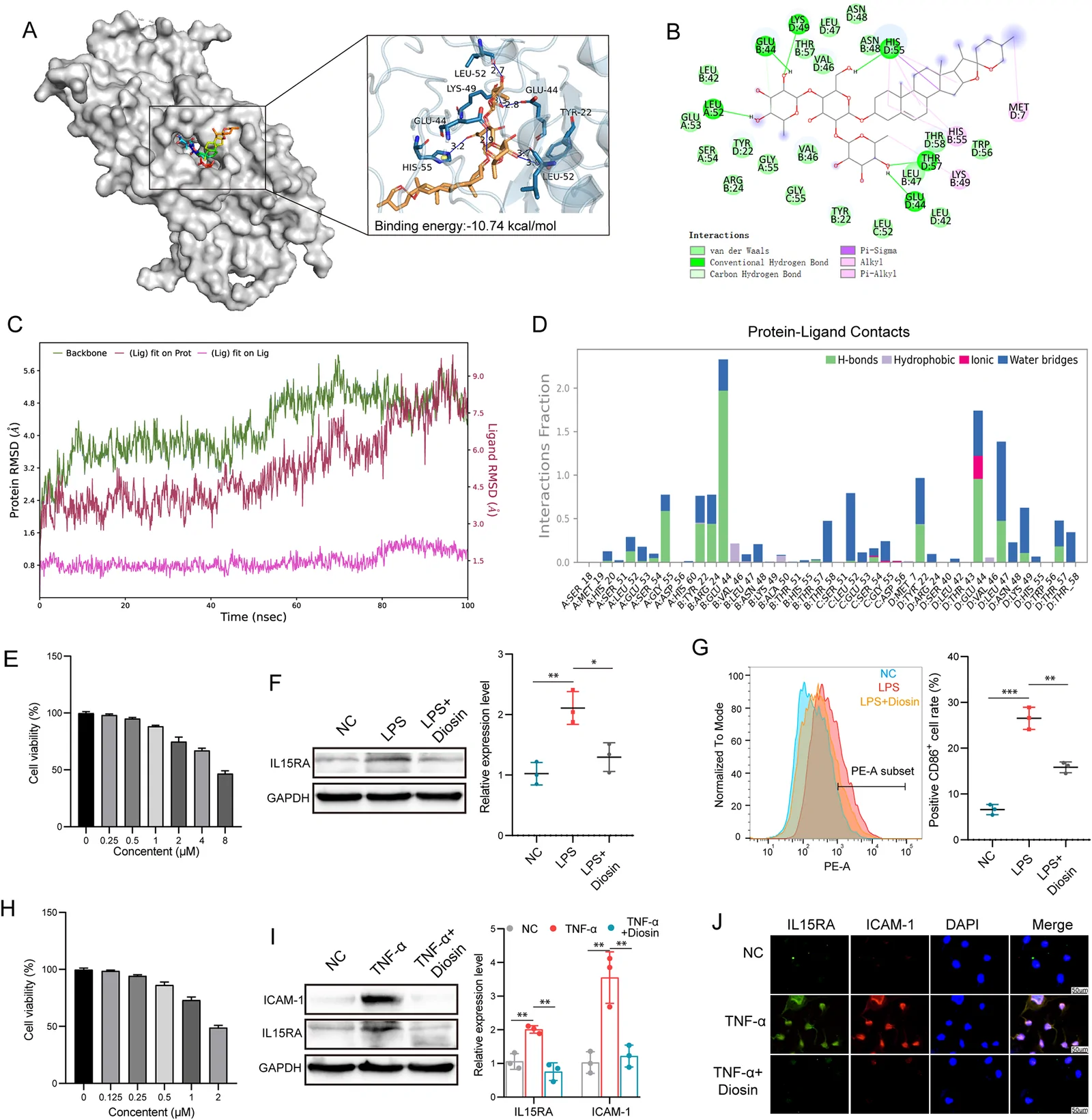
Dioscin inhibits M1 macrophage polarization and endothelial cell inflammation. The 3D **(A)** and 2D **(B)** binding conformations of dioscin and IL15RA. **(C)** RMSD graph showing dioscin interaction at the IL15RA active site for 100 ns. **(D)** Interactions (fraction) of dioscin at the IL15RA active site via MD simulation at 100 ns showing the type of Proteine_Ligand contacts. **(E)** Cells were incubated with increasing concentrations of dioscin for 24 h and viability was evaluated via CCK8 assays. **(F)** The expression of IL15RA in RAW264.7 cells. **(G)** The proportion of CD86^+^ macrophages. **(H)** Cells were incubated with increasing concentrations of dioscin for 24 h and viability was detected via CCK8 assays. **(I)** The expression of IL15RA and ICAM-1 in HUVECs after treatment. **(J)** Immunofluorescence staining for IL15RA, ICAM-1 and DAPI in HUVECs. *p < 0.05, **p < 0.01, ***p < 0.001.

The CCK8 results showed that dioscin effectively decreased the viability of RAW264.7 cells in a dose-dependent manner; and when the concentration of dioscin was greater than 2 μM for 24 h, obvious cytotoxicity was detected (**Figure 7E**). In endothelial cells, 1μM dioscin for 24 h was obviously toxic (**Figure 7H**). To avoid the effects of non-specific toxicity, we treated endothelial cells with 0.5 uM for 24h and macrophages with 1uM for 24h, respectively. Western blot and immunofluorescence analysis revealed that dioscin not only inhibited the expression of IL15RA, but also reduced the proportion of CD86^+^ macrophages (**Figures 7F, G**). In addition, dioscin could significantly inhibit the protein expression levels of IL15RA and ICAM-1 (**Figures 7I, J**). These results indicate that dioscin has certain therapeutic potential for RA and HF by inhibiting the inflammation of macrophages and endothelial cells.

## Discussion

HF is a serious threat to the prognosis of RA patients. And the risk of RA patients developing HF is significantly increased, independent of traditional cardiovascular risk factors (30). The dual challenge of complex drug-disease interactions and the management of comorbidities complicates the disease (31). Therefore, identifying common pathogenic drivers and developing therapeutic strategies that can simultaneously improve RA-associated inflammation and protect cardiac function are urgent clinical needs.

Multi-omics research can provide rapid and effective integrated strategies for therapeutic targets of diseases (32). Through clinical data analysis and Mendelian randomization analysis, we prioritized IL15RA as a key molecular node connecting RA and HF. IL-15, a pleiotropic cytokine that signals through its specific receptor IL15RA, is crucial for the activation and survival of innate and adaptive immune cells, which is directly associated with RA and cardiovascular inflammation (33). In RA, IL-15 is highly expressed in the synovial tissues and fluids. Studies indicate that the IL-15/IL15RA axis is vital for the survival, proliferation, and cytotoxic function of CD8^+^ T cells, which is associated with joint damage (34). The IL-15/IL15RA axis also directly activated endothelial cells, inducing the expression of adhesion molecules such as ICAM-1, thereby promoting leukocyte adhesion, which is a key early event in endothelial dysfunction and atherosclerosis (35). Additionally, IL-15/IL15RA signaling in cardiac macrophages drives pro-inflammatory phenotypes, leading to myocardial inflammation, fibrocyte activation, and adverse ventricular remodeling, all of which are markers of HF progression (36). Our analysis of clinical samples confirmed elevated IL15RA expression in the synovial tissues of RA patients and in the cardiac tissues of CVDs, reinforcing its role as a common pathological factor. Although our cardiac tissue validation was conducted in samples of CVDs rather than in cases of end-stage HF, this difference does not prevent the clinical significance of our findings. Given that CVDs is the main cause of heart failure in RA patients, the upregulation of IL15RA expression observed in the heart tissue of new cardiovascular disease provides supportive evidence for the role of IL15RA in the early inflammatory process that leads to subsequent myocardial dysfunction in RA patients. *In vitro* experiments, inflammation strongly enhances endothelial cells adhesion, and polarizes macrophages to the pro-inflammatory M1 type. Conversely, siIL15RA effectively reversed these phenotypes, reducing endothelial cell activation and the proportion of M1 macrophages. These further revealed that IL15RA modulates the inflammatory activation of endothelial cells and macrophages, which is the central axis of the RA-HF comorbidity. Therefore, as a major regulator of joint destruction and vascular inflammation, inhibiting IL15RA can alleviate the inflammatory cycle between joints and the heart.

In the process of identifying monomers that can inhibit IL15RA, we focused on dioscin, a natural steroid saponin with anti-inflammatory and cardioprotective properties (37). Dioscin can inhibit paw swelling in CIA rats, reduce the thymus index in rats, significantly lower the levels of NF-κB and COX-2, and exhibit significant therapeutic effects on arthritic rats (38). In TNF-α-treated HUVECs and macrophages, dioscin exerts anti-inflammatory effects by inhibiting the NF-κB signaling pathway and the expression of endothelin (ET) (39). Further studies have shown that diosgenin can significantly decrease the apoptosis rate of vascular endothelial cells (ECs), reduce the concentrations of ET and NO, and increase the viability of ECs (40). Batch molecular docking revealed that IL15RA is a plausible candidate for dioscin binding. Cell experiments further confirmed that dioscin effectively inhibited the expression of IL15RA in endothelial cells and macrophages, thereby reducing the expression of ICAM-1 in endothelial cells and the proportion of M1-type macrophages. However, SPR, CETSA and rescue experiments need to be carried out to further prove that the anti-inflammatory effect of dioscin is mediated only by IL15RA. Pending such evidence, our findings indicated that IL15RA is a promising candidate mediator of dioscin’s anti-inflammatory effects, rather than as a validated direct target. This finding indicates that dioscin holds promise as a therapeutic candidate for the management of RA-HF comorbidity. Compared with the independent management of each disease using separate drug regimens, this strategy targeting the common upstream regulator offers a potentially more effective treatment approach for comorbidities.

In conclusion, our study identifies IL15RA as a key molecular mediator linking RA and HF, and positions dioscin—a dietary saponin naturally present in yams (Dioscorea species)—as an IL15RA-modulating candidate with the ability to inhibit this inflammatory pathway. These findings bridge rheumatology, cardiology, and nutritional science, offering a potential nutritional strategy for managing RA-associated cardiac comorbidity.

## Data Availability

The original contributions presented in the study are included in the article/**Supplementary Material**. Further inquiries can be directed to the corresponding authors.
