# Supplementary material for: Integrative population genetics and multi-omics reveal IL15RA as a shared pathogenic hub in rheumatoid arthritis-related heart failure
Source: Front Immunol. 2026 Oct 12;17:1956897. doi: 10.3389/fimmu.2026.1956897 (PMC13696236; doi:10.3389/fimmu.2026.1956897)
Supplement: Supplementary file 1 [file DataSheet1.pdf]

A

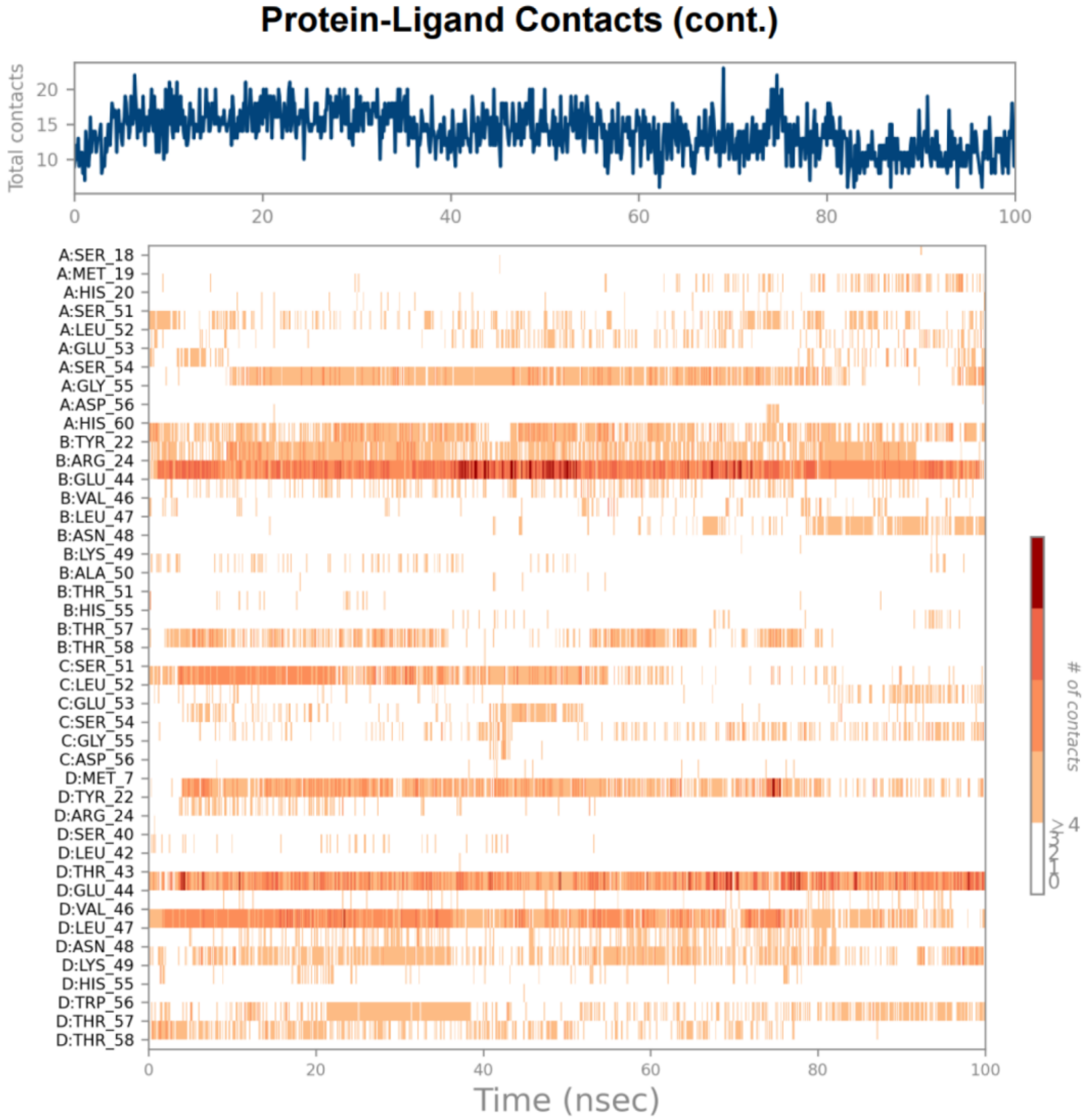

Protein-Ligand Contacts (cont.)
Total contacts
20
15
10
0
20
40
60
80
100
A:SER_18
A:MET_19
A:HIS_20
A:SER_51
A:LEU_52
A:GLU_53
A:SER_54
A:GLY_55
A:ASP_56
A:HIS_60
B:TYR_22
B:ARG_24
B:GLU_44
B:VAL_46
B:LEU_47
B:ASN_48
B:LYS_49
B:ALA_50
B:THR_51
B:HIS_55
B:THR_57
B:THR_58
C:SER_51
C:LEU_52
C:GLU_53
C:SER_54
C:GLY_55
C:ASP_56
D:MET_7
D:TYR_22
D:ARG_24
D:SER_40
D:LEU_42
D:THR_43
D:GLU_44
D:VAL_46
D:LEU_47
D:ASN_48
D:LYS_49
D:HIS_55
D:TRP_56
D:THR_57
D:THR_58
# of contacts
Time (nsec)

B

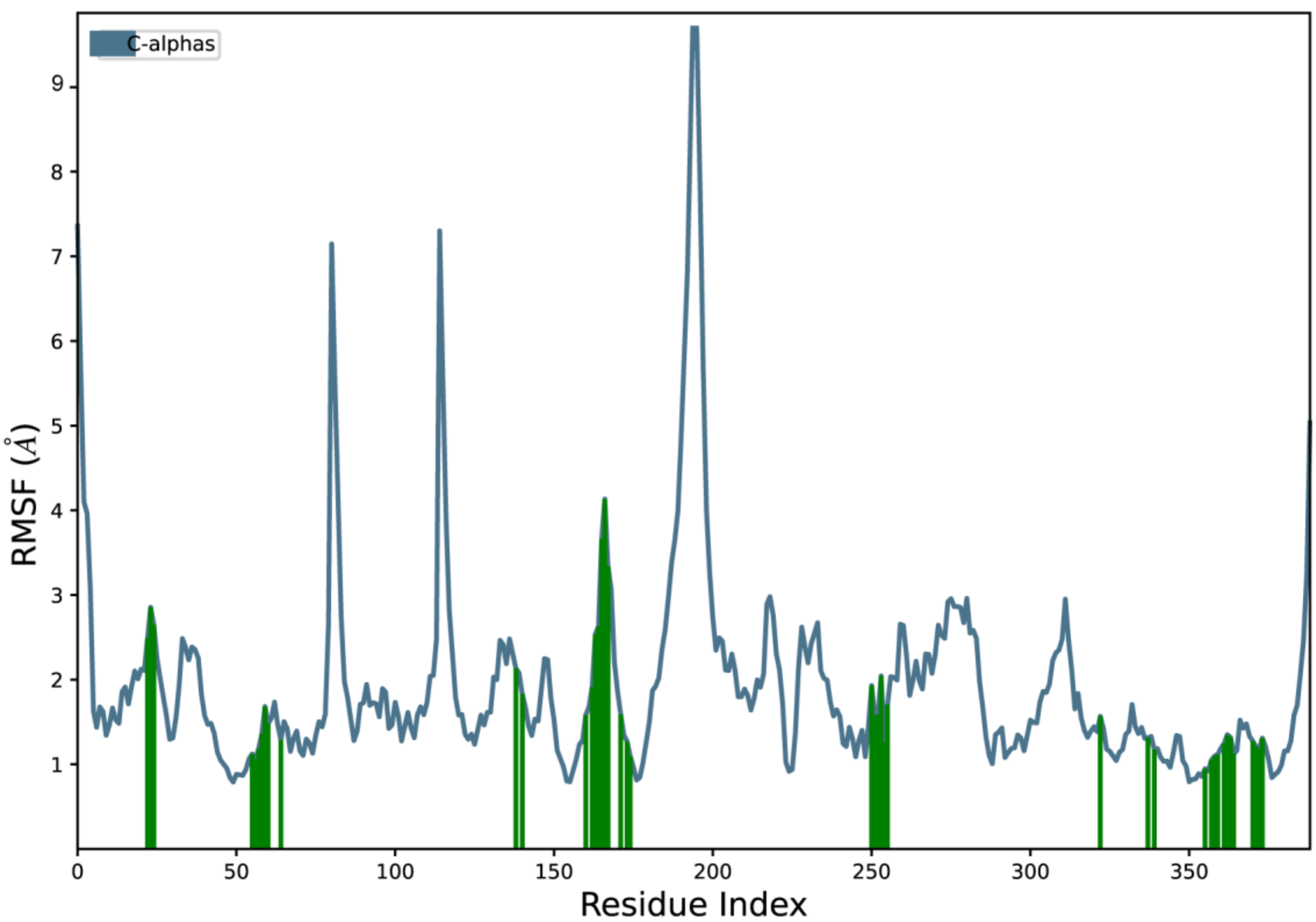

C-alphas
RMSF (Å)
1
2
3
4
5
6
7
8
9
0
50
100
150
200
250
300
350
Residue Index

Supplementary figure S1. Protein-Ligand contacts of dioscin with IL15RA in each trajectory frame. (A) Plot showing the total number of specific contacts the protein makes with the ligand over the course of the trajectory. On the Y-axis, the residues are shown that interact with the ligand in each trajectory time frame (ns) shown on the X-axis. Some residues make more than one specific contact with the ligand, which is represented by a darker shade of orange, according to the scale shown to the right of the plot. (B) RMSF graph of protein IL15RA at 100 ns MD simulation. On this plot, peaks indicate areas of the protein that fluctuate the most during the simulation. Ligand contacts with the protein residues that interact with the ligand are marked with green-coloured vertical bars.

Supplementary Table 1 The sample information of public datasets.

Dataset ID	Platform	Tissue	Control	Disease	Total
GSE12021	GPL96	synovial membrane	19	12	31
GSE55235	GPL96	synovial membrane	20	10	30
GSE55457	GPL96	synovial membrane	20	13	33
GSE55584	GPL96	synovial membrane	6	10	16
GSE89408	GPL1154	synovial membrane	50	152	202
GSE5406	GPL96	myocardium	16	194	210
GSE116250	GPL16791	heart tissue	14	50	64
UKB-a-105		blood	333429	3730	337159
ebi-a-GCST009541		blood	930014	47309	977323
GSE299518	GPL24676	rheumatoid arthritis knee synovium		1	1
GSE222144	human heart	human heart	1	1	2

Supplementary Table 2 The sequences of the primers used for RT_qPCR.

Gene	Forward sequence (5′-3′)	Reverse sequence (5′-3′)	Amplicon Size (bp)
Human			
IL15RA	CCTTCAAAATCACCTTCCAC	GCCTTGACTTGAGGTAGCAT	235
β-actin	CCTTCCTGGGCATGGAGTC	TGATCTTCATTGTGCTGGGTG	189
Mouse			
Il15ra	AGGCCAACTTCCCAGAGTAC	CAGAGGAGCCTTGGAAGACA	181
Gapdh	AAATGGTGAAGGTCGGTGTGAAC	CAACAATCTCCACTTTGCCACTG	206

Supplementary Table 3 Baseline characteristics of study population, NHANES 1999–2018

Characteristics	Heart failure	Non-heart failure	*P*-value
	306 (8.89%)	2625 (91.11%)	
**Age**			
>60	237 (68.8%)	1453 (41.3%)	<0.001
20-60	69 (31.2%)	1172 (58.7%)	
**Gender**			
Male	136 (41.4%)	1056 (40.7%)	0.845

Female	170 (58.6%)	1569 (59.3%)	
**Race**			
Mexican American	36 (4.3%)	426 (6.6%)	<0.01
Non-Hispanic Black	94 (18.1%)	764 (16.6%)	
Non-Hispanic White	150 (73.6%)	1072 (65.8%)	
Other	26 (4%)	363 (11%)	
**Marital status**			
No	178 (49%)	1230 (40.1%)	<0.05
Yes	128 (51%)	1395 (59.9%)	
**Income status**			
Non-Poor	223 (75.9%)	2016 (81.6%)	0.093
Poor	83 (24.1%)	609 (18.4%)	
**BMI**			
<30	169 (51.8%)	1505 (55.6%)	0.323
>=30	137 (48.2%)	1120 (44.4%)	
**Abdominal obesity**			
No	93 (23.5%)	694 (23.7%)	0.963
Yes	213 (76.5%)	1931 (76.3%)	
**Education**			
Below high school	135 (37.8%)	989 (25.8%)	<0.001
High school or above	171 (62.2%)	1636 (74.2%)	
**Smoke**			
No	113 (33.4%)	1211 (42.6%)	<0.01
Yes	193 (66.6%)	1414 (57.4%)	
**Drink**			
No	144 (38.9%)	1192 (38%)	0.827
Yes	162 (61.1%)	1433 (62%)	
**Hypertension**			
No	46 (18.2%)	937 (41.1%)	<0.001
Yes	260 (81.8%)	1688 (58.9%)	
**Diabetes**			
No	179 (64.8%)	2017 (82.2%)	<0.001
Yes	127 (35.2%)	608 (17.8%)	
**Kidney failure**			
No	250 (86.2%)	2457 (94.4%)	<0.001
Yes	56 (13.8%)	168 (5.6%)	
**Stroke**			
No	230 (76.7%)	2421 (93.5%)	<0.001
Yes	76 (23.3%)	204 (6.5%)	

Supplementary Table 4 Odds ratios for heart failure in RA participants with different characteristics

**Characteristics**	**Univariate analysis**	**Multivariate analysis**

	Odds ratio	CI_low	CI_high	*P*-value	Odds ratio	CI_low	CI_high	*P*-value
**Age**								
20-60	Reference							
>60	3.13	2.16	4.52	*P*<0.001	2.5	1.68	3.73	*P*<0.001
**Gender**								
Male								
Female	0.971	0.726	1.3	0.845	0.87	0.63	1.2	0.386
**Race**								
Mexican American	Reference							
Non-Hispanic Black	1.66	0.92	3	0.0945	1.45	0.8	2.62	0.221
Non-Hispanic White	1.7	0.969	2.99	0.0661	2.03	1.15	3.56	0.014
Other	0.549	0.243	1.24	0.15	0.62	0.27	1.44	0.264
**Marital status**								
No	Reference							
Yes	0.696	0.522	0.928	0.0148	0.82	0.58	1.16	0.258
**Income status**								
Poor	Reference							
Non-poor	0.71	0.477	1.06	0.0942	0.68	0.45	1.04	0.075
**BMI**								
<30	Reference							
>=30	1.17	0.86	1.58	0.323	1.22	0.84	1.77	0.292
**Abdominal obesity**								
No	Reference							
Yes	1.01	0.673	1.51	0.963	0.95	0.6	1.53	0.843
**Education**								
Below high school	Reference							
High school or above	0.574	0.425	0.775	*P*<0.001	0.7	0.49	1	0.047
**Smoke**								
No	Reference							
Yes	1.48	1.11	1.97	0.00852	1.49	1.05	2.1	0.025
**Drink**								
No	Reference							
Yes	0.963	0.689	1.35	0.827	1.16	0.76	1.78	0.482
**Hyptersion**								
No	Reference							
Yes	3.14	2.12	4.66	*P*<0.001	1.83	1.18	2.83	0.007
**Diabetes**								

No	Reference							
Yes	2.52	1.8	3.52	*P*<0.001	1.91	1.33	2.73	0.001
**Kidney failure**								
No	Reference							
Yes	2.72	1.79	4.13	*P*<0.001	1.922	1.16	3.18	0.011
**Stroke**								
No	Reference							
Yes	4.36	2.78	6.83	*P*<0.001	3.02	1.87	4.87	*P*<0.001
